# Comprehensive Whole‐Course Management Strategy for Recurrent Renal Cell Carcinoma: Case Report and Literature Review

**DOI:** 10.1002/ccr3.70418

**Published:** 2025-05-01

**Authors:** PengNan Hu, Di Chen, Jun Yu, Hua Mi

**Affiliations:** ^1^ Department of Urology The First Affiliated Hospital of Guangxi Medical University Nanning China

**Keywords:** comprehensive whole‐course management, immunotherapy, molecular targeted therapy, Nivolumab, recurrent renal cell carcinoma

## Abstract

Renal cell carcinoma, a highly recurrent and metastatic malignancy. Drawing upon a decade‐long expertise in managing recurrent cases, we advocate the adoption of Programmed Death‐1 (PD‐1) inhibitors in conjunction with Axitinib when monotherapy with Axitinib fails to restrain tumor metastasis, thereby empowering doctors with an efficacious treatment option.

## 1 Introduction

As a prevalent malignancy affecting the urinary system, renal cell carcinoma (RCC) comprises approximately 3%–5% of adult malignant tumors and accounts for 85% of renal malignant tumors. It is the second most common malignancy in the male urinary system, following prostate and bladder cancer [1, 2]. RCC exhibits a high propensity for metastasis, with 25% of patients presenting with metastatic disease at the initial diagnosis and an additional 50% developing metastasis during follow‐up [3]. The recurrence of RCC poses significant challenges to both patients and clinicians. Timely treatment of tumor recurrence and prevention of metastasis can be achieved through long‐term and effective comprehensive management of renal cancer patients. In this study, we present a 10‐year comprehensive management approach for a patient with recurrent RCC and review relevant literature to explore a comprehensive whole‐course management strategy for RCC patients.

## 2 Case History

**FIGURE 1:**
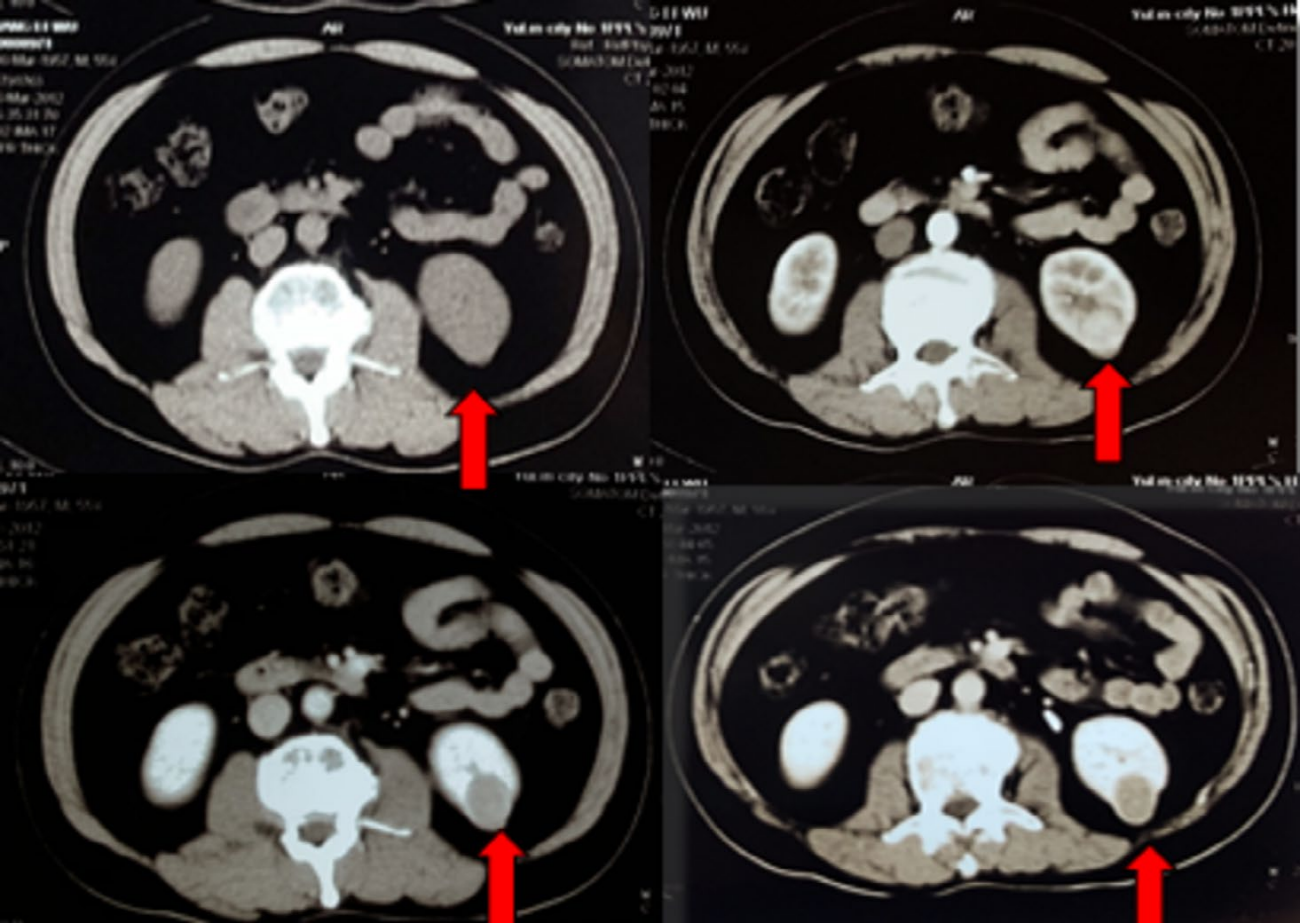
Urinary CT examination on first admission.

A 62‐year‐old male patient was admitted to the hospital in February 2012 due to “Left renal mass noted on imaging a month ago.” Physical examination showed no abnormalities, and computed tomography (CT) urogram revealed a space‐occupying lesion measuring approximately 3.0 × 2.5 × 2.0 cm in the middle dorsal part of the left kidney (Figure 1). The preoperative diagnosis was a left renal tumor (stage I, cT1aN0M0). Laparoscopic partial nephrectomy was performed, and postoperative pathology confirmed clear cell carcinoma of the left kidney, Fuhrman grade III. The patient had a smooth recovery and was discharged as planned. Regular follow‐up examinations including chest X‐ray, ultrasound of urinary tract or CT urogram were done every 3 months for the first 2 years and then every 6 months for the next 3 years. No adjuvant treatments such as chemotherapy, radiotherapy, or immunotherapy were administered.

## 3 Investigation and Treatment

**FIGURE 2:**
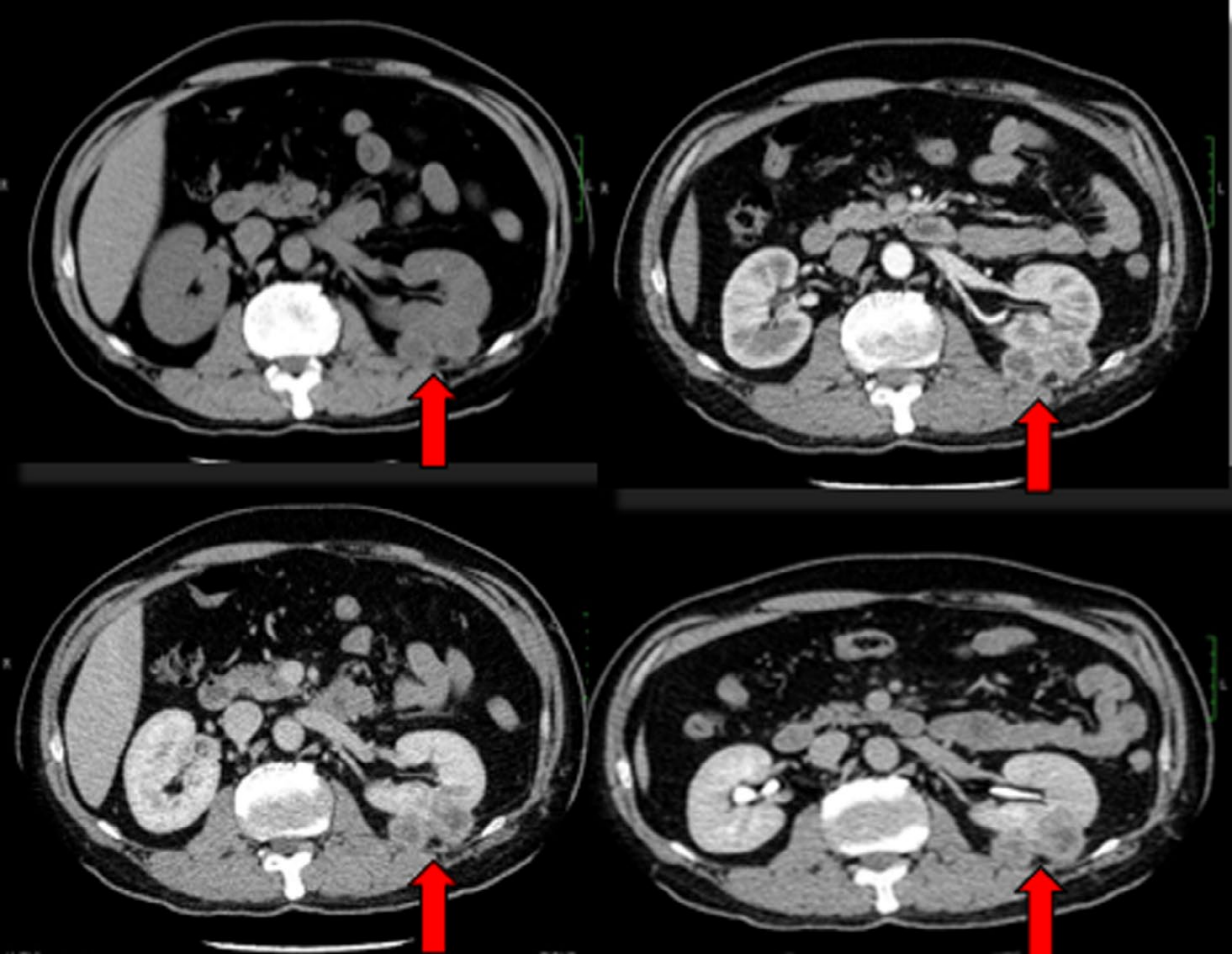
Urinary CT examination on second admission.

In April 2015, the patient was readmitted to the hospital due to a recurrence of a left renal tumor that was discovered during a follow‐up CT urogram. The imaging showed that the tumor had locally recurred and measured 3.2 × 2.5 cm in size (Figure 2). Based on the preoperative examination, the patient was diagnosed with locally advanced RCC, specifically left renal clear cell carcinoma (rT3N0M0 stage III). A laparoscopic radical resection of the left RCC was successfully performed. During the surgery, it was observed that the tumor had infiltrated the perirenal fascia. The postoperative staging was switched to left renal clear cell carcinoma (rT4N0M0, stage IV).

Following radical nephrectomy for left RCC, the patient began treatment with Sunitinib (50 mg daily for 4 weeks followed by 2 weeks break) in July 2015. Unfortunately, the patient experienced side effects and was diagnosed with grade 3 hand‐foot syndrome. Despite switching to a 2 weeks on, 1 week off regimen, the patient was unable to tolerate the medication and stopped taking it on his own.

**FIGURE 3:**
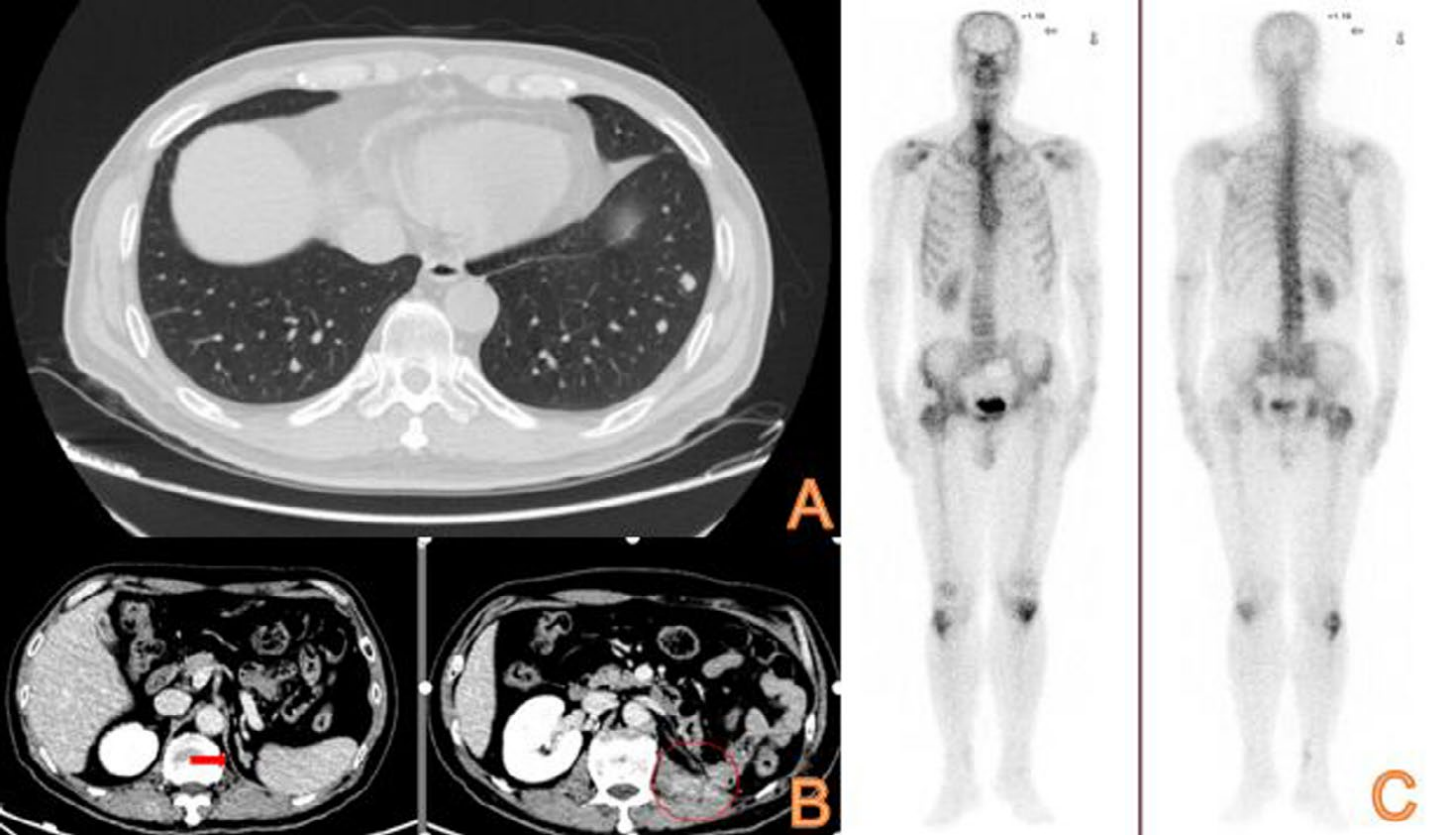
(A) Chest CT reveals lung metastases in May 2016. (B) Urinary CT reveals left adrenal metastasis in October 2017. (C) whole‐body radionuclide bone scan reveals metastatic lesions in the right acetabulum and upper femur in October 2016.

In May 2016, a chest CT examination revealed the presence of multiple nodules in both lungs, which were determined to be metastatic tumors originating from renal cancer (Figure 3). Subsequently, the patient underwent a thoracoscopic biopsy of the right lung mass, and the postoperative pathology confirmed the presence of metastatic clear cell carcinoma. Due to the patient's previous intolerance to Sunitinib, a second‐line molecular targeted therapy with Axitinib (5 mg, twice daily) was initiated in June 2016.

**FIGURE 4:**
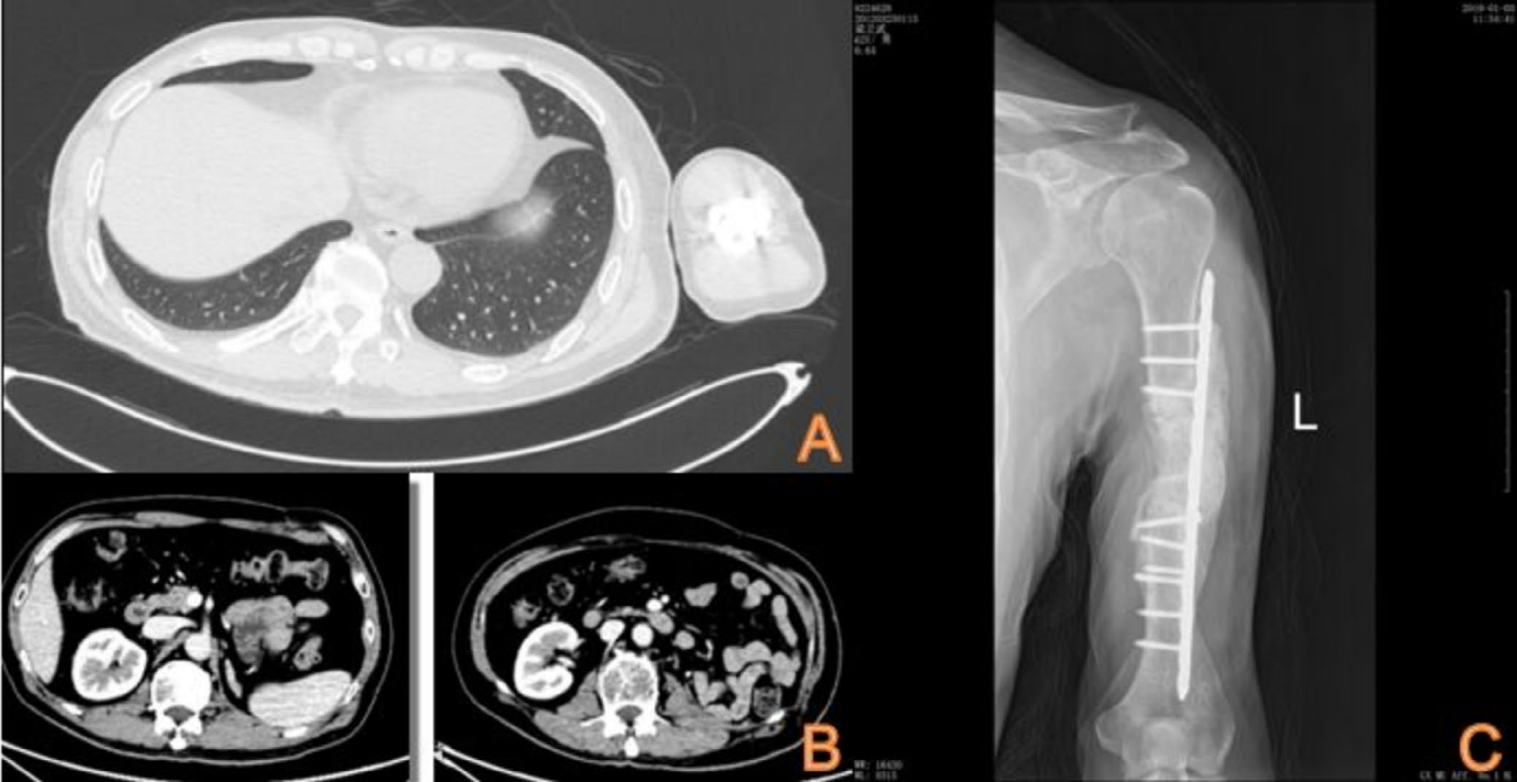
(A) Follow‐up chest CT shows significantly decreased lung metastases in May 2019. (B) Follow‐up urinary CT reveals decreased size of left adrenal metastasis in March 2018. (C) Left humerus X‐ray review shows no local bone metastatic recurrence and good humerus fixation in January 2019.

In October 2016, the patient developed pain and immobility in their right lower limb. An X‐ray examination revealed bone destruction in the right femur, while radionuclides bone scan showed bone metastasis in the right acetabulum and upper femur (Figure 4). Subsequently, in November 2016, an X‐ray confirmed the presence of a metastatic lesion in the right fibula. These findings indicated the progression to clinical stage IV RCC, prompting surgical resection of the metastatic lesion. In November 2016, the patient underwent bone tumor resection of the right proximal femur and right fibular mass resection. This was followed by left knee joint mass resection in March 2017 and left humerus tumor resection in August 2017. Pathology reports from all four operations confirmed the presence of metastatic clear cell carcinoma. Furthermore, in January 2017, the patient's dosage of Axitinib was increased to 7.5 mg twice daily.

**FIGURE 5:**
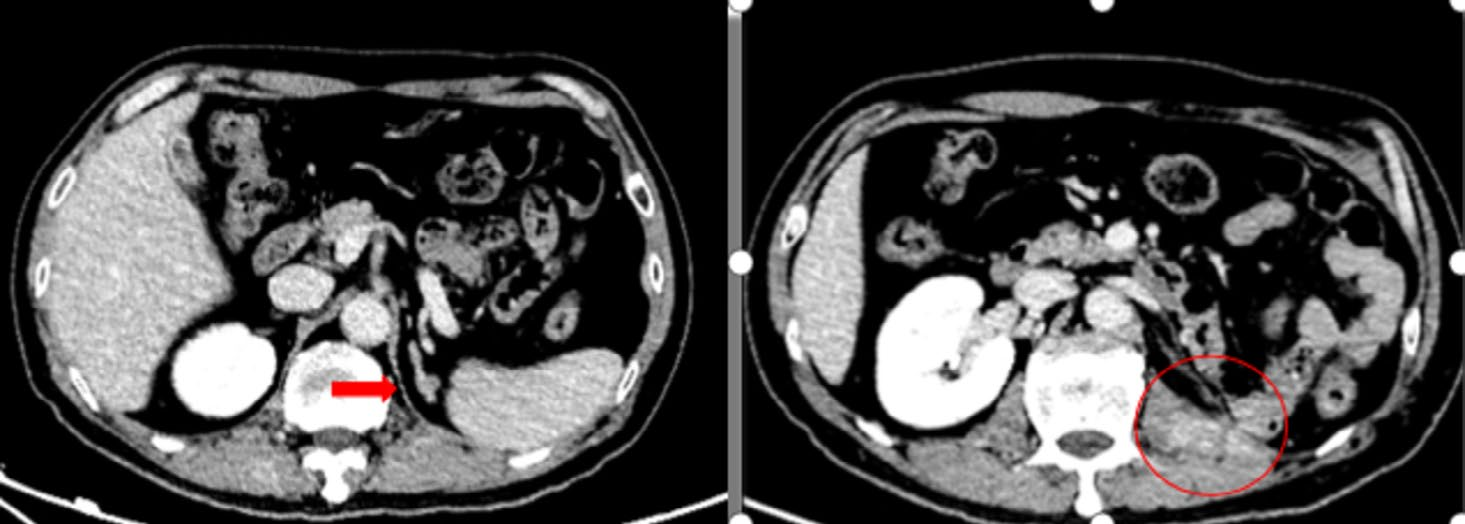
Urinary CT in October 2017.

In October 2017, a follow‐up CT scan of the urinary system revealed the presence of a left adrenal nodule and enhanced left psoas major muscle (Figure 5). These findings were indicative of adrenal metastasis from RCC. As a result, in November 2017, the patient's treatment plan was adjusted to include a combination of Axitinib and Nivolumab (3 mg/kg dose administered weekly).

## 4 Results and Follow‐Up

**FIGURE 6:**
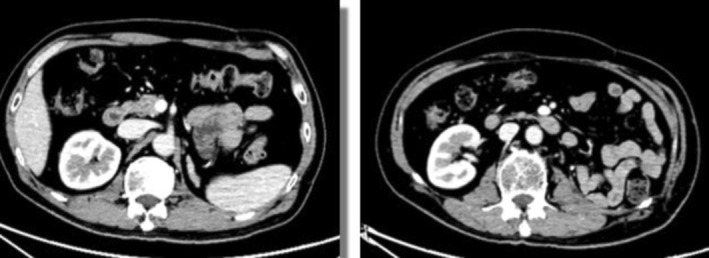
Urinary CT was reexamined in March 2018.

**FIGURE 7:**
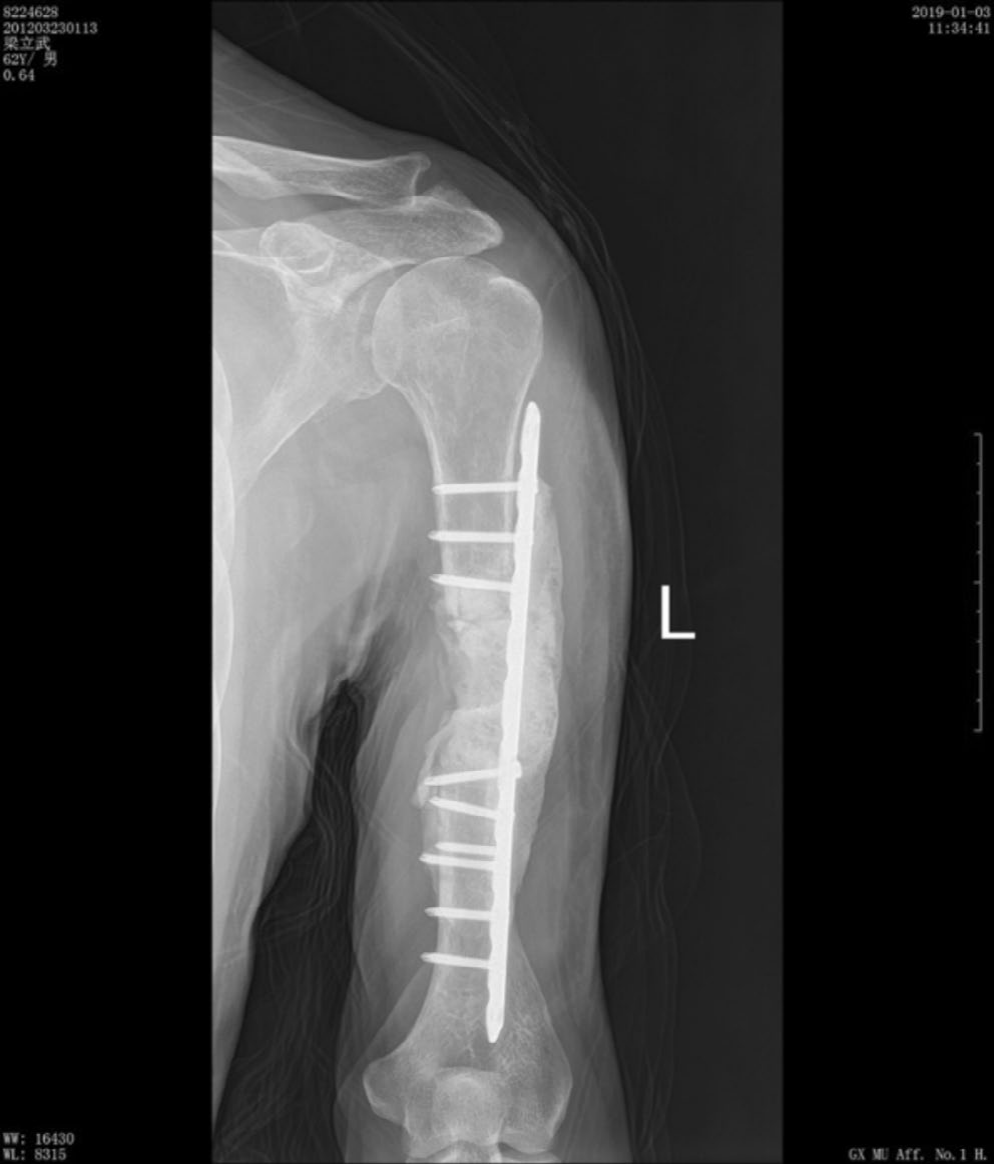
X‐ray examination of the left humerus was performed in January 2019.

**FIGURE 8:**
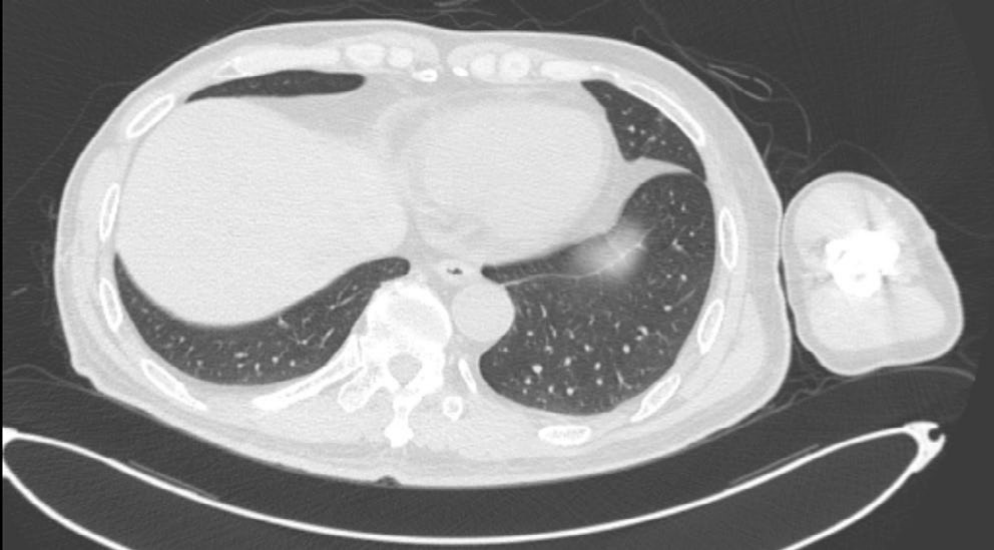
Chest CT in May 2019.

In March 2018, a follow‐up CT urogram revealed a reduction in the size of the adrenal lesion (Figure 6). Additionally, an X‐ray examination of the left humerus in January 2019 showed no visible signs of metastatic lesions (Figure 7). In May 2019, chest CT showed that pulmonary lesions were less visible (Figure 8). Most recently, in March 2022, chest, abdomen, and pelvic CT did not reveal any recurrence of metastatic lesions. As of now, the patient is in good condition, able to walk with crutches, and has experienced no drug side effects. The patient is continuing the same regimen, which includes axitinib combined with Nivolumab, administered at the previously established dose and frequency.

## 5 Time Line

- 2012.02 A left renal tumor was identified and treated with laparoscopic partial nephrectomy (PN).
- 2015.04 The tumor recurred in the primary left renal area and was treated with laparoscopic radical resection.
- 2015.07 Sunitinib treatment (50 mg qd) was discontinued due to adverse reactions.
- 2016.05 Lung metastases were detected and treated with Axitinib (5 mg twice daily).
- 2016.11 The right proximal femur bone tumor and right fibula mass were excised.
- 2017.01 Axitinib dose therapy (7.5 mg twice daily).
- 2017.03 The left knee joint mass was excised.
- 2017.08 Left humerus tumor resection was performed.
- 2017.10 Left adrenal metastasis was found.
- 2017.11 Treatment involved Axitinib (7.5 mg twice daily) combined with Nivolumab (3 mg/kg, once weekly).
- 2018.03 Reexamination revealed a reduced left adrenal metastasis.
- 2019.05 CT examination of the chest, abdomen, and urinary system showed reduced metastasis in the lungs and the left adrenal gland.
- 2022.03 CT examination of the chest, abdomen, and pelvis showed no obvious metastasis.

## 6 Discussion

Recurrent of RCC refers to the reemergence of tumors in areas such as the residual kidney, renal fossa, ipsilateral adrenal gland, and distant organs like the lungs and bones. This recurrence can occur after treatments such as PN, radical nephrectomy (RN), or ablation therapy. It is estimated that approximately 20% to 40% of patients with RCC will experience tumor recurrence and metastasis following surgery [4, 5].

Axitinib is a targeted therapy medication, specifically an anti‐tumor agent designed to eliminate tumor cells by precisely targeting defined sites within the tumor. In this case, the patient's disease worsened with Axitinib monotherapy. However, combining it with Nivolumab marked a turning point, suppressing metastatic lesions and preventing new ones. Axitinib is an indazole derivative that is a selective inhibitor of Vascular Endothelial Growth Factors(VEGFR)‐1, 2, and 3. Compared with tyrosine kinase receptor antagonists such as Sorafenib, Sunitinib, and Pazopanib, Axitinib showed higher selectivity for VEGFR [6, 7]. On the other hand, Nivolumab, a human IgG4 Programmed Death‐1 (PD‐1) immune checkpoint inhibitor, disrupts Programmed Death‐Ligand 1 (PD‐L1) mediated signaling to provide anti‐tumor therapy [8]. It is recommended in the 2017 NCCN guidelines for disease progression after molecular targeted therapy [9]. Systemic Nivolumab administration can elicit durable therapeutic responses not seen with conventional cytotoxic anticancer drugs. Due to RCC being highly vascular and PD‐L1 being highly expressed in cancer cells, numerous studies propose combining PD‐1 inhibitors with VEGF/VEGFR inhibitors (TKI) as a feasible therapy to prevent RCC recurrence [10, 11, 12]. This regimen combines the inhibition of the highly vascular nature of RCC by TKI with the action of PD‐1 inhibitors, which represents a new direction for the treatment of patients with relapsed RCC. For example, a meta‐analysis by Umberto Capitanio et al. demonstrated that patients receiving Pembrolizumab‐Lenvatinib or Pembrolizumab‐Axitinib had superior overall survival (OS), progression‐free survival (PFS), and duration of response compared to those receiving Sunitinib alone [13]. This finding serves as level 1a evidence supporting the use of Pembrolizumab combined with TKI as a first‐line treatment for advanced RCC. Rini BI et al. reported a randomized phase III trial in advanced RCC, in which Axitinib plus Pembrolizumab, compared with Sunitinib, showed superior OS, PFS, and objective response rate (ORR) [14]. According to the 2023 NCCN guidelines [12], the combination of Cabozantinib and Nivolumab is considered a preferred category 1 treatment option for clear cell renal cell carcinoma (ccRCC). In some studies, this combination therapy has demonstrated even stronger efficacy than Pembrolizumab combined with TKI. For instance, Bradley McGregor et al. reported that Nivolumab plus Cabozantinib significantly improved PFS, ORR, and duration of response compared to Pembrolizumab combined with Axitinib [15]. In the 2024 NCCN guidelines, Axitinib and Nivolumab are also recommended as category 1 options for sequentially treating patients with recurrent or surgically unresectable phase IV ccRCC [16]. This aligns with the treatment received by the patient featured in this article. Consequently, it perfectly clarifies why the patient experienced a reduction in metastatic lesions and no recurrence under the current treatment plan.

Our findings suggest that sequential therapy combining PD‐1 inhibitors with Axitinib is a feasible approach to effectively suppress RCC recurrence in cases of persistent tumor recurrence and metastasis.

A well‐crafted follow‐up plan is indispensable for the management of recurrent RCC. The patient described in this article adhered to a rigorous follow‐up protocol, which included chest X‐ray, ultrasound of the urinary tract, and CT urogram every 3 months for the initial 2 years post‐surgery, followed by a six‐monthly schedule for the subsequent 3 years. This regimen is consonant with the guidelines established by the European Association of Urology (EAU) in 2012, which advise imaging examinations—such as chest X‐ray, ultrasound of the urinary tract, or CT urogram—every 3–6 months for 2 years after surgery for patients with locally advanced or high‐risk RCC [17]. Additionally, this protocol incorporates the expertise of regional clinicians. Considering that the patient's pathology was ccRCC, a subtype associated with a high risk of recurrence, we conducted meticulous follow‐ups every 3 months for the first 2 years post‐surgery. This approach provided a solid foundation for the successful detection of recurrent RCC and any metastatic spread.

Of course, our treatment program has shortcomings in assessing patient prognosis and adverse drug reactions. In the current study, various methods assess the impact of ICI on the prognosis and health of kidney cancer patients. The Royal Marsden Hospital Score evaluates patient prognosis based on laboratory tests and clinical features. It serves as an assessment tool for treatment efficacy and prognosis with the patient in this paper, enabling timely treatment strategy adjustments [18]. Recent years have seen increased focus on quality of life (QoL) assessment in tumor patient treatment. Some systematic reviews reveal that QoL assessment is often excluded from phase II or III renal cancer clinical trial protocols. It might lead to overlooking the patient's current psychological and physical health, thereby potentially affecting prognosis [19]. Another study indicated that ICI may cause hearing damage; yet the patient in this paper did not provide feedback on this issue. Thus, assessing for hearing damage can be a crucial aspect of our QoL assessment. At the same time, we should also use FKSI‐DRS, EQ5D, and EORTC QLQ‐C30 scores to evaluate the quality of life of the patient, facilitating prompt treatment of potential psychological and health issues [20].

## 7 Conclusions

In this presentation, we report on our expertise in the comprehensive management of recurrent RCC spanning a decade. Our conclusions indicate that rigorous, long‐term postoperative monitoring, interdisciplinary teamwork, and tailored therapeutic strategies grounded in the pathophysiological underpinnings of the cancer constitute effective approaches for the successful management of patients diagnosed with recurrent RCC.

## Author Contributions

**PengNan Hu:** conceptualization, data curation, investigation, writing – original draft, writing – review and editing. **Di Chen:** methodology, writing – original draft. **Jun Yu:** conceptualization, investigation, writing – original draft. **Hua Mi:** conceptualization, data curation, investigation, project administration, supervision, writing – review and editing.

## Ethics Statement

The 62‐year‐old patient involved in this study, after thoroughly understanding the details of the research and obtaining adequate informed consent, voluntarily agreed to participate in our research, granting us access to their hospitalization records and personal information. All procedures performed in this study were in accordance with the ethical standards of the Helsinki Declaration (as revised in 2013). The study was approved by the First Affiliated Hospital of Guangxi Medical University ethics board (No. 2024‐E051‐01).

## Consent

Written informed consent was obtained from the patient to publish this report in accordance with the journal's patient consent policy.

## Conflicts of Interest

The authors declare no conflicts of interest.

## Data Availability

The data that support the findings of this study are available from the corresponding author upon reasonable request.
